# Relationship of METS-IR with cardiometabolic multimorbidity in China: a nationwide longitudinal cohort study

**DOI:** 10.3389/fnut.2025.1518840

**Published:** 2025-02-12

**Authors:** Chunyan Zhou, Yanyu Zhang, Xiaoyi Liu, Chenyu He, Shiyang Li

**Affiliations:** ^1^Department of Geriatrics, Panzhihua Central Hospital, Panzhihua, China; ^2^Clinical Laboratory, Panzhihua Central Hospital, Panzhihua, China; ^3^Panzhihua Central Hospital Affiliated to Dali University, Dali, Yunnan, China

**Keywords:** cardiometabolic multimorbidity, METS-IR, cardiometabolic disease, CHARLS, risk

## Abstract

**Background:**

Cardiometabolic multimorbidity (CMM) has emerged as a global health challenge with a high mortality risk. This study aimed to explore the association between the metabolic score for insulin resistance (METS-IR) and the incidence of CMM.

**Methods:**

This study included 6,977 individuals in the CHARLS database. We used multiple cox proportional hazards regression and restricted cubic splines (RCS) analysis to evaluate the association between METS-IR and CMM. Subgroup analyses and interaction tests were also performed.

**Results:**

During a median 109 (108–109) months of follow-up, 745 (10.7%) participants were diagnosed with new-onset CMM. The incidences of CMM among participants in quartiles (Q) 1–4 of METS-IR were 4.99, 7.51, 10.67, and 19.54%, respectively. METS-IR was significantly higher in individuals with CMM compared to those without CMM (*p* < 0.001). After multivariate adjustment, a higher METS-IR was significantly associated with an increased risk of CMM. Compared to participants in Q1 of METS-IR, the hazard ratios (HRs) (95% confidence intervals [CIs]) using cox proportional hazards regression analysis for those in Q2–4 were 1.52 (1.15–2.00), 2.02 (1.56–2.63), and 3.61 (2.80–4.64), respectively. RCS analysis revealed a significant nonlinear association between METS-IR and CMM (nonlinear *p* < 0.05). The association between METS-IR and the incidence of CMM was present in almost all the subgroups. Furthermore, the predictive ability of METS-IR for CMM was 0.669, which surpassed that of both the triglyceride to high-density lipoprotein cholesterol ratio and the triglyceride glucose index.

**Conclusion:**

A higher METS-IR was closely associated with an increased risk of CMM. Further studies on METS-IR could be beneficial for preventing and treating CMM.

## Introduction

Cardiometabolic multimorbidity (CMM), defined as the simultaneous presence of at least two cardiometabolic diseases, including heart disease, diabetes, and stroke, constitutes one of the most common and severe multimorbidity profiles (1, 2). Previous study has reported that individuals with CMM exhibited nearly a two-fold higher risk of all-cause mortality compared to those with individual cardiometabolic diseases (CMD) (3). Additionally, CMM has been demonstrated to be associated with frailty (4), cognitive decline (5), and even dementia (6). Over the past few decades, the prevalence of CMM has significantly increased due to the rise in life expectancy and various risk factors associated with cardiovascular diseases, imposing substantial health and economic burdens on both society and individuals (6, 7). However, previous studies have primarily focused on individual CMD, rarely exploring this complex condition in its entirety. Given the severity of CMM and its rapidly increasing incidence globally (8), early prediction of CMM is crucial for timely diagnosis and treatment, aiming to reduce the adverse outcomes.

Insulin resistance (IR), characterized by a diminished response to insulin in target cells, has been reported to be associated with various diseases, including hypertension, diabetes, and coronary artery disease (9, 10). The hyperinsulinemic euglycemic clamp (HEC) has long been considered as the gold standard for assessing insulin resistance (11). However, its invasive, expensive, and complex nature limits its applicability in large-scale clinical and epidemiological studies (12, 13). Additionally, other insulin-based IR metrics are also constrained in practical application due to concerns regarding their accuracy and stability (12, 14). The metabolic score for insulin resistance (METS-IR), a novel surrogate indicator of insulin resistance, has gained increasing attention in recent years (14). In a cross-sectional study involving 1,576 participants without cardiovascular disease, increased METS-IR was correlated with a higher prevalence of coronary artery calcification (15). Su et al. found that METS-IR was positively associated with the risk of heart failure (16). A cohort study by Duan et al. indicated that METS-IR was a significant predictor of all-cause and cardiovascular mortality in the U.S. population (17). Additionally, METS-IR was demonstrated to be independently and positively associated with the risk of prediabetes in the Chinese population (18). However, these studies have primarily focused on the relationships between METS-IR and individual CMD. To date, no research has assessed the association between METS-IR and CMM using a prospective cohort. In the present study, we performed a longitudinal analysis using data from the China Health and Retirement Longitudinal Study (CHARLS) to evaluate the predictive value of METS-IR for the risk of developing CMM among the Chinese population.

## Materials and methods

### Study population

**Figure 1:**
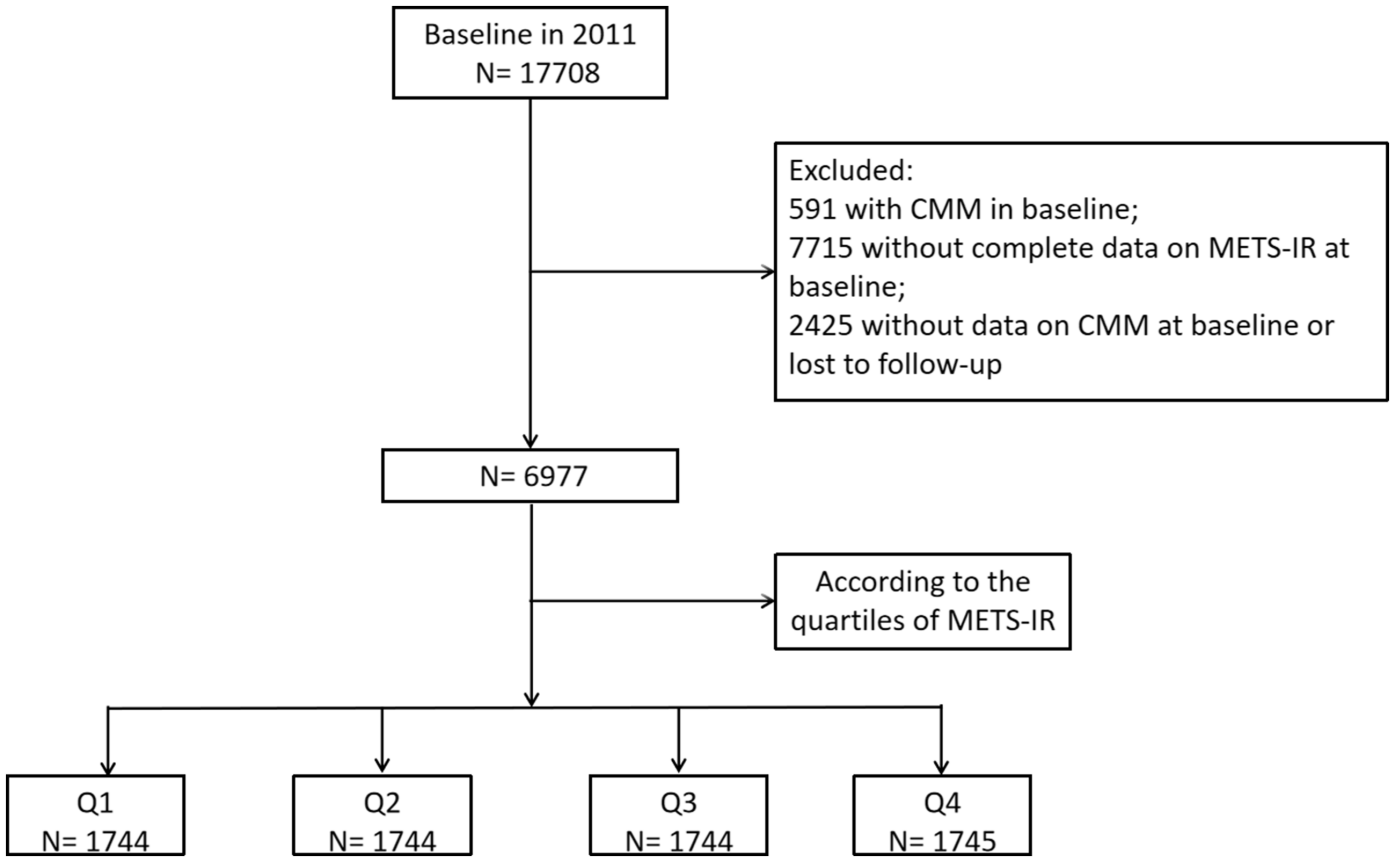
Flow chart of sample selection and the exclusion criteria.

Study participants were drawn from the China Health and Retirement Longitudinal Study (CHARLS), which is a nationally representative longitudinal survey of residents in rural and urban areas of China of ≥45 years old that commenced in 2011 and their spouse. Detailed information regarding the study design and enrollment criteria have been previously reported (19). The study consists of five waves of surveys conducted between 2011 and 2020, with participants recruited from 23 Chinese provinces using a multistage stratified probability proportional-to-size sampling strategy. The national baseline survey was conducted in 2011, enrolling 17,708 participants, followed by four subsequent waves: Wave 2 (2013–2014), Wave 3 (2015–2016), Wave 4 (2017–2018), and Wave 5 (2019–2020). We applied data from wave 1–5, which are available online at http://charls.pku.edu.cn. The inclusion criteria encompassed individuals aged 18 years or older who had completed follow-up data, including complete data on CMM and METS-IR. The exclusion criteria included: (1) participants with CMM in Wave 1; (2) individuals without complete data on METS-IR at baseline; and (3) Missing data on CMM at baseline or lost to follow-up. Ultimately, a total of 6,977 individuals met the eligibility criteria for subsequent analysis (Figure 1). The original CHARLS study was approved by the Ethical Review Committee of Peking University (IRB00001052-11015), and written informed consent was obtained from all participants at the time of enrollment.

### Assessment of CMM events

CMM events were defined as the simultaneous presence of at least two cardiometabolic diseases, including diabetes, heart disease and stroke. The diagnoses of heart disease and stroke were confirmed through self-report of a physician’s diagnosis obtained via the questionnaire survey, including “Have you been told by a doctor that you have been diagnosed with a heart attack, Angina, coronary heart disease, heart failure, or other heart problems?” or “Have you been told by a doctor that you have been diagnosed with a stroke?” (20). In addition to self-reporting diabetes, participants were diagnosed with diabetes if they met any of the following criteria: (1) fasting plasma glucose ≥ 7.0 mmol/L; (2) random plasma glucose ≥ 11.1 mmol/L; or (3) HbA1c ≥ 6.5% according to the American Diabetes Association criteria (21). The incidence of CMM was determined at the time of diagnosis of the second CMD, at which point the individuals presented with two distinct types of CMD.

### Assessment of METS-IR and other covariates

In this study, extensive baseline information was collected by trained.

interviewers following standard procedures. Anthropometric variables including the height (m) and weight (kg) were measured by trained medical staff according to standard protocol. The body mass index (BMI) was calculated as weight divided by height squared (kg/m^2^). After at least 5 min spent sitting down, the blood pressure of the participants was measured three times by a trained interviewer at 45-s intervals using a digital sphygmomanometer (Omron TM HEM-7200). Blood samples were collected after an overnight fast, then stored at −20°C and transported to Beijing for further measurements. The biochemical parameters included white blood cell count (WBC), platelet count (PLT), hemoglobin, fasting blood glucose (FBG), uric acid (UA), serum creatinine, Cystatin C, high-sensitivity C-reactive protein (hsCRP), and lipid profiles. The METS-IR were calculated according to the following formula: METS-IR = ln [2 × FPG (mg/dL) + fasting serum TG (mg/dL)] × BMI (kg/m^2^)/ln [HDL − C (mg/dL)] (22).

All participants provided medical history and lifestyle information during face-to-face interviews by trained interviewers. A structured questionnaire was employed to collect the baseline data, including age, gender, hypertension, marital status (married and others), residence (rural, urban), education (elementary school and below, secondary school, and college and above), smoking status, and drinking status. Smoking status was categorized as “never smoking,” “current smoker” and “former smoker.” Participants were diagnosed with hypertension if their systolic blood pressure was ≥140 mmHg, diastolic blood pressure was ≥90 mmHg, or they had a self-reported history of hypertension, or were currently using antihypertensive medications.

### Statistical analysis

Continuous variables were presented as the mean ± standard deviation or the median with interquartile range, depending on the normality of the data distribution. Categorical variables were presented as frequency and percentage. For comparison of variables between CMM group and non-CMM group, independent t-test was used for normally distributed data and Man-Whitney test were for skewed data. One-way analyses of variance (for normally distributed data), Kruskal-Wallis tests (for skewed data), and chi-square tests (for categorical data) were performed to assess the differences between more than two groups. In our study, the population was divided into four groups according to the quartiles of baseline METS-IR as follows: Q1 (<29.6), Q2 (≥29.6, <34.2), Q3 (≥34.2, <40.0), and Q4 (≥40.0). The Kaplan–Meier analysis was used to assess the cumulative incidence of CMM among METS-IR quartiles, and the differences of curves were examined utilizing the log-rank test. Cox proportional hazards regression was used to estimate the hazard ratios (HRs) and 95% confidence intervals (95% CIs) between METS-IR quartiles and incidence of CMM. Prior to conducting the Cox regression model, we evaluated the proportional hazards assumption using Schoenfeld residuals and found no potential violations. The dose–response relationship between METS-IR and the risk of developing CMM was investigated through restricted cubic splines (RCS) analysis. Subgroup analyses were performed to explore whether the association between METS-IR with CMM differed by gender, age, hypertension, residence, marital status, education, smoking status, and drinking status. Receiver operating characteristic (ROC) curve analysis was used to evaluate the predictive ability of variables for CMM and Delong’s test was used to compare the differences between variables.

All statistical analyses in the present study were performed using RStudio 4.2.1 software, SPSS 26, and an online statistical analysis platform called I STATISTICS.^1^ All comparisons were two-sided, and a significance level of *p* < 0.05 was considered statistically significant.

## Results

### Participants characteristics

Figure 1 depicts the overall methodological workflow of the present study. A total of 6,977 participants were included for analysis according to the inclusion and exclusion criteria. During a median 109 (108–109) months of follow-up, 745 (10.7%) participants were identified with new-onset CMM. Compared to individuals without CMM, those with CMM were older, had a higher proportion of females, and exhibited elevated levels of systolic blood pressure (SBP), diastolic blood pressure (DBP), BMI, WBC, PLT, hemoglobin, FBG, total cholesterol (TC), triglycerides (TG), low-density lipoprotein cholesterol (LDL-C), UA, hsCRP, METS-IR, as well as a higher incidence of hypertension, basal diabetes, basal stroke, and basal heart disease (*p* < 0.05) (Table 1). Conversely, individuals in this group had a lower prevalence of current smoking and drinking and exhibited reduced level of high-density lipoprotein cholesterol (HDL-C) (*p* < 0.05).

**Table 1:** Baseline characteristics of the study participants according to cardiometabolic multimorbidity.

Characteristics	Overall	Non-CMM group	CMM group	*p*-value
Participants, no	6,977	6,232	745	
Age, years	58.75 ± 9.61	58.54 ± 9.71	60.49 ± 8.61	<0.001
Male, *n* (%)	3,171 (45.45)	2,878 (46.18)	293 (39.33)	<0.001
SBP, mmHg	128.64 ± 21.17	127.74 ± 20.82	136.21 ± 22.57	<0.001
DBP, mmHg	74.98 ± 12.06	74.64 ± 12.00	77.83 ± 12.17	<0.001
**Residence, *n* (%)**				0.142
Rural	4,644 (66.56)	4,166 (66.85)	478 (64.16)	
Urban	2,333 (33.44)	2,066 (33.15)	267 (35.84)	
Marriage, married, *n* (%)	6,151 (88.16)	5,500 (88.25)	651 (87.38)	0.486
**Educational level, *n* (%)**				0.174
Primary	4,953 (70.99)	4,406 (70.70)	547 (73.42)	
Secondary	1,388 (19.89)	1,259 (20.20)	129 (17.32)	
Third	636 (9.12)	567 (9.10)	69 (9.26)	
**Smoking, *n* (%)**				<0.001
Never	4,291 (61.50)	3,791 (60.83)	500 (67.11)	
Former	582 (8.34)	504 (8.09)	78 (10.47)	
Current	2,104 (30.16)	1,937 (31.08)	167 (22.42)	
Current drinking, *n* (%)	2,297 (32.92)	2,096 (33.63)	201 (26.98)	<0.001
Hypertension, *n* (%)	3,246 (46.52)	2,740 (43.97)	506 (67.92)	<0.001
Basal diabetes, *n* (%)	885 (12.68)	682 (10.94)	203 (27.25)	<0.001
Basal stroke, *n* (%)	110 (1.58)	77 (1.24)	33 (4.43)	<0.001
Basal heart disease, *n* (%)	648 (9.29)	449 (7.20)	199 (26.71)	<0.001
BMI, kg/m^2^	23.05 (20.85–25.67)	22.88 (20.70–25.39)	24.95 (22.33–27.55)	<0.001
WBC, 10^9^/L	6.00 (4.97–7.20)	5.95 (4.90–7.20)	6.20 (5.10–7.50)	0.002
PLT, 10^9^/L	213.31 ± 76.40	212.07 ± 72.29	223.66 ± 104.15	0.003
Hemoglobin, g/dL	14.35 ± 2.22	14.32 ± 2.22	14.55 ± 2.26	0.01
FBG, mg/dL	102.06 (94.32–112.50)	101.52 (93.96–111.42)	108.72 (99.00–123.66)	<0.001
TC, mg/dL	193.35 ± 38.41	192.44 ± 38.12	200.94 ± 40.00	<0.001
TG, mg/dL	103.54 (74.34–152.22)	101.78 (73.46–148.68)	124.79 (88.50–182.31)	<0.001
LDL-C, mg/dL	116.69 ± 34.86	115.94 ± 34.31	122.97 ± 38.62	<0.001
HDL-C, mg/dL	51.33 ± 15.12	51.81 ± 15.09	47.26 ± 14.70	<0.001
UA, mg/dL	4.40 ± 1.24	4.38 ± 1.23	4.54 ± 1.30	0.001
Serum creatinine, mg/dL	0.78 ± 0.24	0.78 ± 0.24	0.79 ± 0.22	0.184
Cystatin C, mg/L	0.98 (0.86–1.13)	0.98 (0.86–1.12)	0.99 (0.86–1.16)	0.125
hsCRP, mg/L	0.99 (0.54–2.13)	0.96 (0.53–2.04)	1.36 (0.68–2.60)	<0.001
METS-IR	34.15 (29.61–39.96)	33.72 (29.35–39.17)	39.17 (33.04–45.19)	<0.001

SBP, systolic blood pressure; DBP, diastolic blood pressure; BMI, body mass index; WBC, white blood cell count; PLT, platelet count; FBG, fasting blood glucose; TC, total cholesterol; TG, triglycerides; LDL-C, low-density lipoprotein cholesterol; HDL-C, high-density lipoprotein cholesterol; UA, Uric acid; hsCRP, high-sensitivity C-reactive protein; CMM, cardiometabolic multimorbidity; METS-IR, Metabolic score for insulin resistance.

Normal variables were reported as mean ± SD, non-normal variables were reported as median (Q1–Q2).

We also compared the baseline characteristics of the included and lost individuals. Supplementary Table S1 showed that participants who were lost to follow-up were older, more likely to be male and live in urban, less likely to be married and educated, exhibited higher levels of SBP, DBP, FBG, UA, serum creatinine, hsCRP, and had higher prevalence of basal stroke, and basal heart disease (*p* < 0.05). Conversely, individuals in this group had a lower prevalence of current smoking and drinking, and exhibited reduced level of BMI and hemoglobin (*p* < 0.05).

### Baseline characteristics based on the quantiles of METS-IR

The participants were divided into four groups based on the quartiles of baseline METS-IR (Table 2). The incidence of CMM in quartiles Q1, Q2, Q3, and Q4 were 4.99, 7.51, 10.67, and 19.54%, respectively. Participants in the higher METS-IR quantile groups were younger, more likely to be female, and exhibited higher levels of SBP, DBP, BMI, WBC, PLT, hemoglobin, FBG, TC, TG, LDL-C, UA, and hsCRP (*p* < 0.05). Additionally, individuals in the higher METS-IR quantile groups were more likely to reside in urban areas and to be married, while also exhibiting higher levels of education and a greater proportion of hypertension, basal diabetes, and basal heart disease (*p* < 0.05). In contrast, the levels of HDL-C, Cystatin C, and the prevalence of current smoking and drinking were lower in the higher METS-IR quantile groups (*p* < 0.05).

**Table 2:** Baseline characteristics of participants according to the quartiles of METS-IR.

Characteristics	METS-IR				*p*-value
	Q1 (<29.6)	Q2 (≥29.6, <34.2)	Q3 (≥34.2, <40.0)	Q4 (≥40.0)	
Participants, no	1,744	1,744	1,744	1,745	
Age, years	61.32 ± 10.13	58.38 ± 9.81	58.12 ± 9.07	57.17 ± 8.89	<0.001
Male, *n* (%)	909 (52.12)	832 (47.71)	745 (42.72)	685 (39.26)	<0.001
SBP, mmHg	125.10 ± 21.67	126.08 ± 20.38	129.75 ± 20.78	133.66 ± 20.76	<0.001
DBP, mmHg	71.89 ± 11.91	73.30 ± 11.34	75.85 ± 11.83	78.91 ± 11.95	<0.001
**Residence, *n* (%)**					<0.001
Rural	1,327 (76.09)	1,247 (71.50)	1,089 (62.44)	981 (56.22)	
Urban	417 (23.91)	497 (28.50)	655 (37.56)	764 (43.78)	
Marriage, married, *n* (%)	1,465 (84.00)	1,539 (88.25)	1,545 (88.59)	1,602 (91.81)	<0.001
**Educational level, *n* (%)**					<0.001
Primary	1,368 (78.44)	1,259 (72.19)	1,183 (67.83)	1,143 (65.50)	
Secondary	266 (15.25)	339 (19.44)	374 (21.44)	409 (23.44)	
Third	110 (6.31)	146 (8.37)	187 (10.72)	193 (11.06)	
**Smoking, *n* (%)**					<0.001
Never	920 (52.75)	1,044 (59.86)	1,130 (64.79)	44.69 (42.10,48.58)	
Former	137 (7.86)	131 (7.51)	137 (7.86)	177 (10.14)	
Current	687 (39.39)	569 (32.63)	477 (27.35)	371 (21.26)	
Current drinking, *n* (%)	667 (38.25)	599 (34.35)	560 (32.11)	471 (26.99)	<0.001
Hypertension, *n* (%)	640 (36.70)	685 (39.28)	858 (49.20)	1,063 (60.92)	<0.001
Basal diabetes, *n* (%)	89 (5.10)	163 (9.35)	217 (12.44)	416 (23.84)	<0.001
Basal stroke, *n* (%)	29 (1.66)	20 (1.15)	29 (1.66)	32 (1.83)	0.394
Basal heart disease, *n* (%)	141 (8.08)	134 (7.68)	171 (9.81)	202 (11.58)	<0.001
BMI, kg/m^2^	19.63 (18.36–20.73)	22.18 (21.17–23.18)	24.28 (23.06–25.55)	27.39 (25.81–29.27)	<0.001
WBC, 10^9^/L	5.76 (4.70–6.92)	5.90 (4.90–7.20)	6.00 (5.00–7.30)	6.20 (5.20–7.50)	<0.001
PLT, 10^9^/L	209.54 ± 74.62	209.61 ± 71.56	215.19 ± 74.60	218.87 ± 83.92	<0.001
Hemoglobin, g/dL	13.97 ± 2.10	14.31 ± 2.29	14.39 ± 2.11	14.73 ± 2.33	<0.001
FBG, mg/dL	98.28 (91.26–106.02)	100.08 (93.06–109.71)	102.78 (95.58–112.68)	109.08 (99.27–124.20)	<0.001
TC, mg/dL	190.88 ± 35.94	189.62 ± 36.81	193.86 ± 39.07	199.02 ± 40.98	<0.001
TG, mg/dL	76.11 (58.41–97.57)	90.27 (69.03–122.13)	113.28 (84.96–155.76)	164.61 (118.59–240.72)	<0.001
LDL-C, mg/dL	112.19 ± 31.78	116.69 ± 32.48	121.18 ± 34.48	116.70 ± 39.62	<0.001
HDL-C, mg/dL	64.54 ± 15.08	54.20 ± 11.49	47.44 ± 10.34	39.13 ± 10.06	<0.001
UA, mg/dL	4.27 ± 1.20	4.26 ± 1.18	4.44 ± 1.24	4.64 ± 1.28	<0.001
Serum creatinine, mg/dL	0.78 ± 0.23	0.77 ± 0.32	0.78 ± 0.18	0.78 ± 0.21	0.735
Cystatin C, mg/L	1.01 (0.89–1.17)	0.98 (0.87–1.12)	0.97 (0.85–1.12)	0.95 (0.81–1.09)	<0.001
hsCRP, mg/L	0.73 (0.43–1.61)	0.81 (0.47–1.75)	1.04 (0.58–2.09)	1.46 (0.81–2.88)	<0.001
METS-IR	27.04 (25.10–28.48)	31.89 (30.76–32.98)	36.76 (35.39–38.19)	44.69 (42.10–48.58)	<0.001
CMM, *n* (%)	87 (4.99)	131 (7.51)	186 (10.67)	341 (19.54)	<0.001

SBP, systolic blood pressure; DBP, diastolic blood pressure; BMI, body mass index; WBC, white blood cell count; PLT, platelet count; FBG, fasting blood glucose; TC, total cholesterol; TG, triglycerides; LDL-C, low-density lipoprotein cholesterol; HDL-C, high-density lipoprotein cholesterol; UA, Uric acid; hsCRP, high-sensitivity C-reactive protein; CMM, cardiometabolic multimorbidity; METS-IR, Metabolic score for insulin resistance.

### Relationship between METS-IR and the incidence of CMM

**Figure 2:**
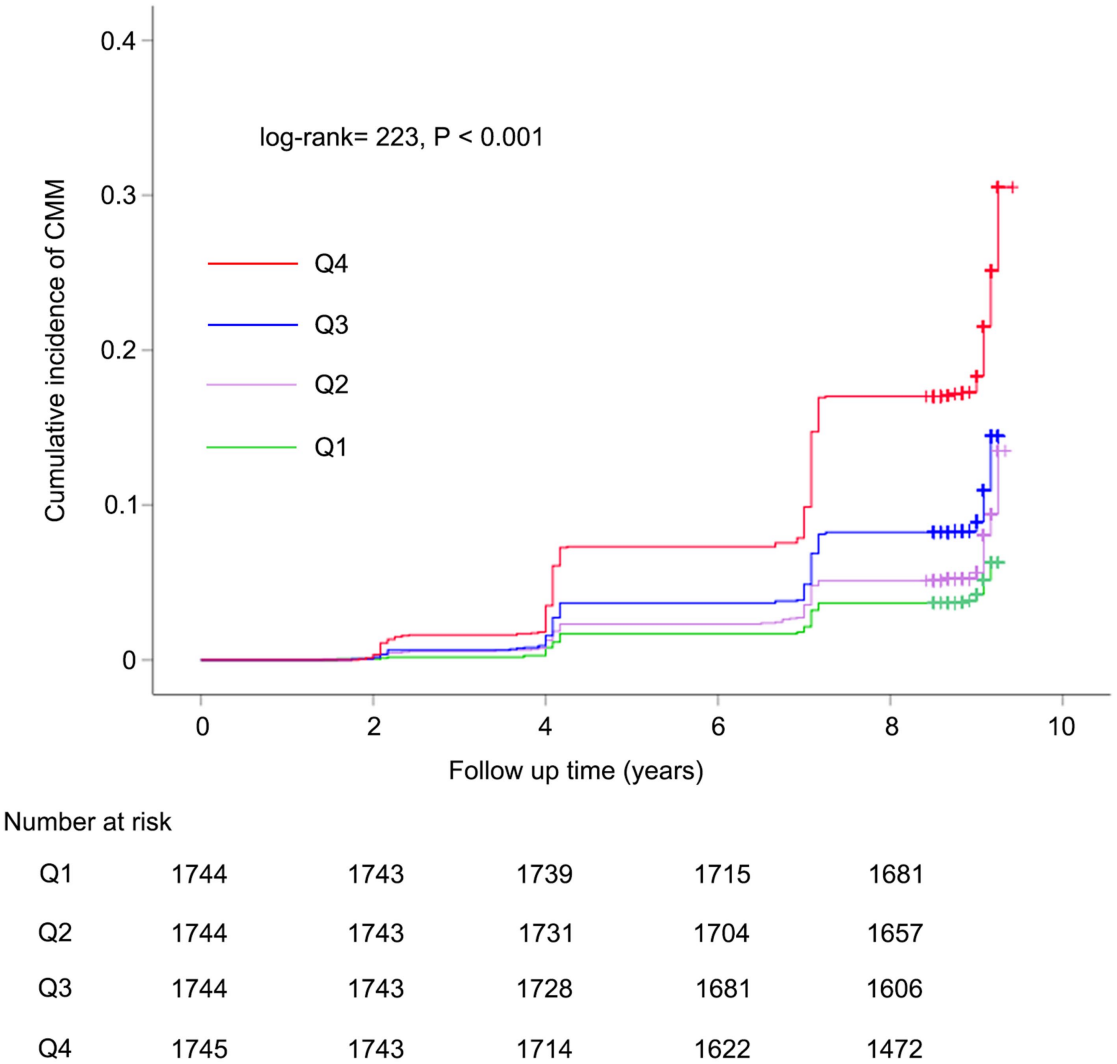
Kaplan–Meier curves for the cumulative incidence of CMM. CMM, cardiometabolic multimorbidity.

Kaplan–Meier survival curve was utilized to analyze the cumulative incidence of CMM. Figure 2 illustrates that the cumulative incidence of CMM increased gradually with increasing METS-IR across the quartiles (log-rank = 223, *p* < 0.001). Subsequently, we performed multicollinearity analysis and determined that the variance inflation factor (VIF) for all variables (including METS-IR, age, gender, hypertension, smoking status, drinking status, rural residence, marital status, education, SBP, DBP, WBC, PLT, hemoglobin, serum creatinine, and hsCRP) were less than 5 (Supplementary Table S2), indicating the absence of collinearity among these variables. We assessed the proportional hazards assumption using the Schoenfeld residual test and found no violations (*p* > 0.05). Table 3 summarizes the association between METS-IR quantile and the risk of CMM using Cox proportional hazards regression model analysis. Model 1 was an unadjusted model; Model 2 adjusted for age, gender; Model 3 further adjusted for hypertension, smoking status, drinking status, rural residence, marital status, education, SBP, DBP, WBC, PLT, hemoglobin, serum creatinine, and hsCRP. Among participants without CMM at baseline, multi-adjusted Cox regression models revealed that the HRs and 95% CIs of CMM were 1.52 (1.15–2.00) for participants in Q2, 2.02 (1.56–2.63) for those in Q3, and 3.61 (2.80–4.64) for those in Q4, compared to participants in Q1 (Table 3). We subsequently categorized the population into four groups based on the types of CMD: individuals without any CMDs at baseline, those with heart disease at baseline, those with diabetes at baseline, and those with stroke at baseline. Table 3 showed that METS-IR quantiles were significantly associated with the risk of CMM, regardless of adjustment for other confounding factors in the group without any CMDs at baseline, consistent with the results obtained prior to grouping. Additionally, a significant correlation between METS-IR quantile and CMM was also observed in groups with heart disease (Q4 vs. Q1: HR = 2.54, 95% CI = 1.55–4.14, *p* < 0.001; Q3 vs. Q1: HR = 1.82, 95% CI = 1.12–2.96, *p* = 0.016) and diabetes (Q4 vs. Q1: HR = 2.21, 95% CI = 1.17–4.19, *p* = 0.015) at baseline.

**Table 3:** Cox proportional hazards regression model analysis of baseline METS-IR and CMM.

		Model 1		Model 2		Model 3	
	METS-IR, Categories	HR (95%CI)	*p*-value	HR (95%CI)	*p*-value	HR (95%CI)	*p*-value
Without CMM at baseline	Q1	Reference		Reference		Reference	
	Q2	1.50 (1.14–1.96)	0.004	1.61 (1.23–2.11)	<0.001	1.52 (1.15–2.00)	0.003
	Q3	2.16 (1.67–2.78)	<0.001	2.32 (1.80–3.01)	<0.001	2.02 (1.56–2.63)	<0.001
	Q4	4.14 (3.27–5.23)	<0.001	4.61 (3.63–5.85)	<0.001	3.61 (2.80–4.64)	<0.001
Without any CMD at baseline	Q1	Reference		Reference		Reference	
	Q2	1.65 (1.14–2.38)	0.008	1.80 (1.24–2.61)	0.002	1.73 (1.19–2.52)	0.004
	Q3	1.98 (1.38–2.84)	<0.001	2.22 (1.54–3.19)	<0.001	2.03 (1.40–2.95)	<0.001
	Q4	3.19 (2.26–4.51)	<0.001	3.71 (2.61–5.28)	<0.001	3.16 (2.18–4.59)	<0.001
With heart disease at baseline	Q1	Reference		Reference		Reference	
	Q2	1.09 (0.64–1.87)	0.752	1.12 (0.65–1.93)	0.671	1.14 (0.65–1.97)	0.648
	Q3	1.90 (1.20–3.02)	0.007	1.90 (1.19–3.03)	0.007	1.82 (1.12–2.96)	0.016
	Q4	2.80 (1.81–4.34)	<0.001	2.84 (1.82–4.43)	<0.001	2.54 (1.55–4.14)	<0.001
With diabetes at baseline	Q1	Reference		Reference		Reference	
	Q2	1.27 (0.62–2.58)	0.509	1.25 (0.61–2.54)	0.546	1.24 (0.61–2.55)	0.551
	Q3	1.46 (0.74–2.85)	0.272	1.41 (0.72–2.78)	0.317	1.21 (0.61–2.41)	0.586
	Q4	2.74 (1.48–5.08)	0.001	2.74 (1.47–5.11)	0.002	2.21 (1.17–4.19)	0.015
With stroke at baseline	Q1	Reference		Reference		Reference	
	Q2	1.92 (0.52–7.15)	0.331	2.04 (0.55–7.58)	0.289	1.74 (0.42–7.26)	0.45
	Q3	2.56 (0.79–8.33)	0.117	2.92 (0.89–9.63)	0.078	2.45 (0.61–9.81)	0.205
	Q4	4.12 (1.36–12.44)	0.012	4.65 (1.50–14.41)	0.008	2.50 (0.64–9.68)	0.186

CMM, cardiometabolic multimorbidity; METS-IR, Metabolic score for insulin resistance; CMD, cardiometabolic disease; HR, hazard ratio; CI, confidence interval.

Model 1 was an unadjusted model; Model 2 adjusted for age, gender; Model 3 further adjusted for hypertension, smoking status, drinking status, rural residence, marital status, education, SBP, DBP, WBC, PLT, hemoglobin, serum creatinine, and hsCRP.

**Figure 3:**
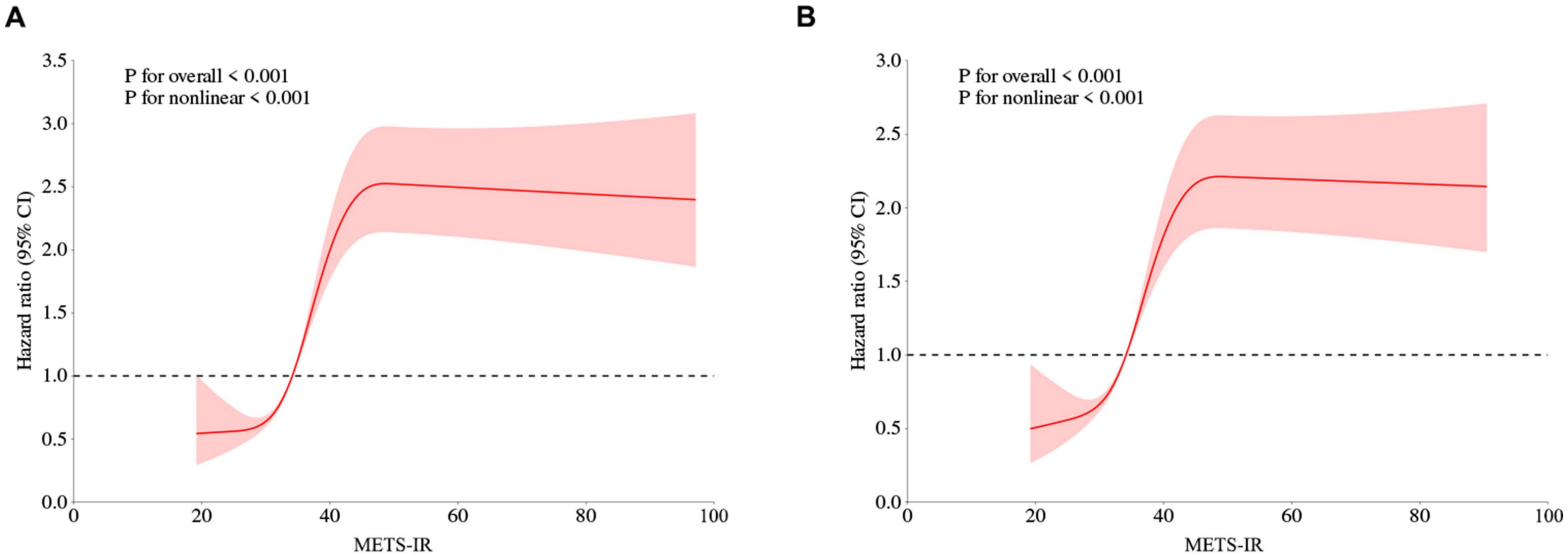
Restricted cubic spline curves for CMM according to METS-IR using Cox proportional hazards regression model analysis without **(A)** or with **(B)** adjustment for other confounding factors. METS-IR, Metabolic score for insulin resistance; CMM, cardiometabolic multimorbidity.

Restricted cubic spline is a flexible and powerful method for data fitting, especially when dealing with time-varying covariant effects or non-proportional risks. Conversely, fractional polynomials may be overly restrictive, often assuming linear or logarithmic time relationships, which might oversimplify and fail to capture complex data variations. Therefore, we performed RCS to model and visualize the relationship between METS-IR and the risk of CMM. Figure 3 presented a positive nonlinear association between METS-IR and the risk of CMM, even after adjusting for confounding covariates including age, gender, hypertension, smoking status, drinking status, rural residence, marital status, education, SBP, DBP, WBC, PLT, hemoglobin, serum creatinine, and hsCRP (nonlinear *p* < 0.05).

### Subgroup analysis

**Figure 4:**
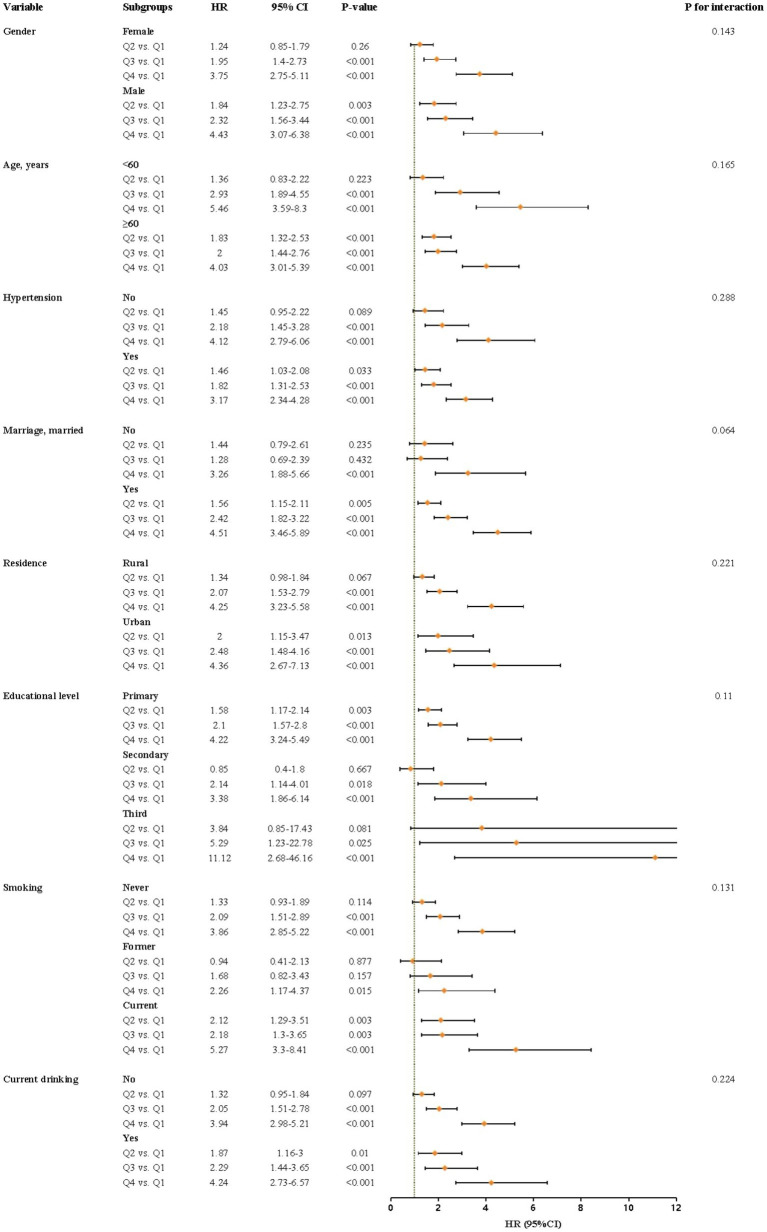
Subgroup analysis of the relationship between METS-IR and the risk of CMM using cox proportional hazards regression model. CMM, cardiometabolic multimorbidity; HR, hazard ratios; CI, Confidence interval.

We performed weighted interaction tests and subgroup analyses to assess whether the association between METS-IR and the risk of developing CMM differs across different subgroups. The result showed that individuals in quartiles Q3 and Q4 were associated with the risk of developing CMM across all subgroups, except for individuals who are not married and former smokers (Figure 4), when compared to those in quartiles Q1. Additionally, the difference in the risk of developing CMM between Q2 and Q1 was statistically significant in subgroups of male, individuals with age ≥ 60 years, without hypertension, be married, urban residence, with primary education, current smoking and drinking (*p* < 0.05). Furthermore, we observed no significant interactive effect between these subgroups and METS-IR on the risk of CMM in Cox proportional hazards regression analysis (*p* for interaction > 0.05).

### Discriminative power analysis

**Figure 5:**
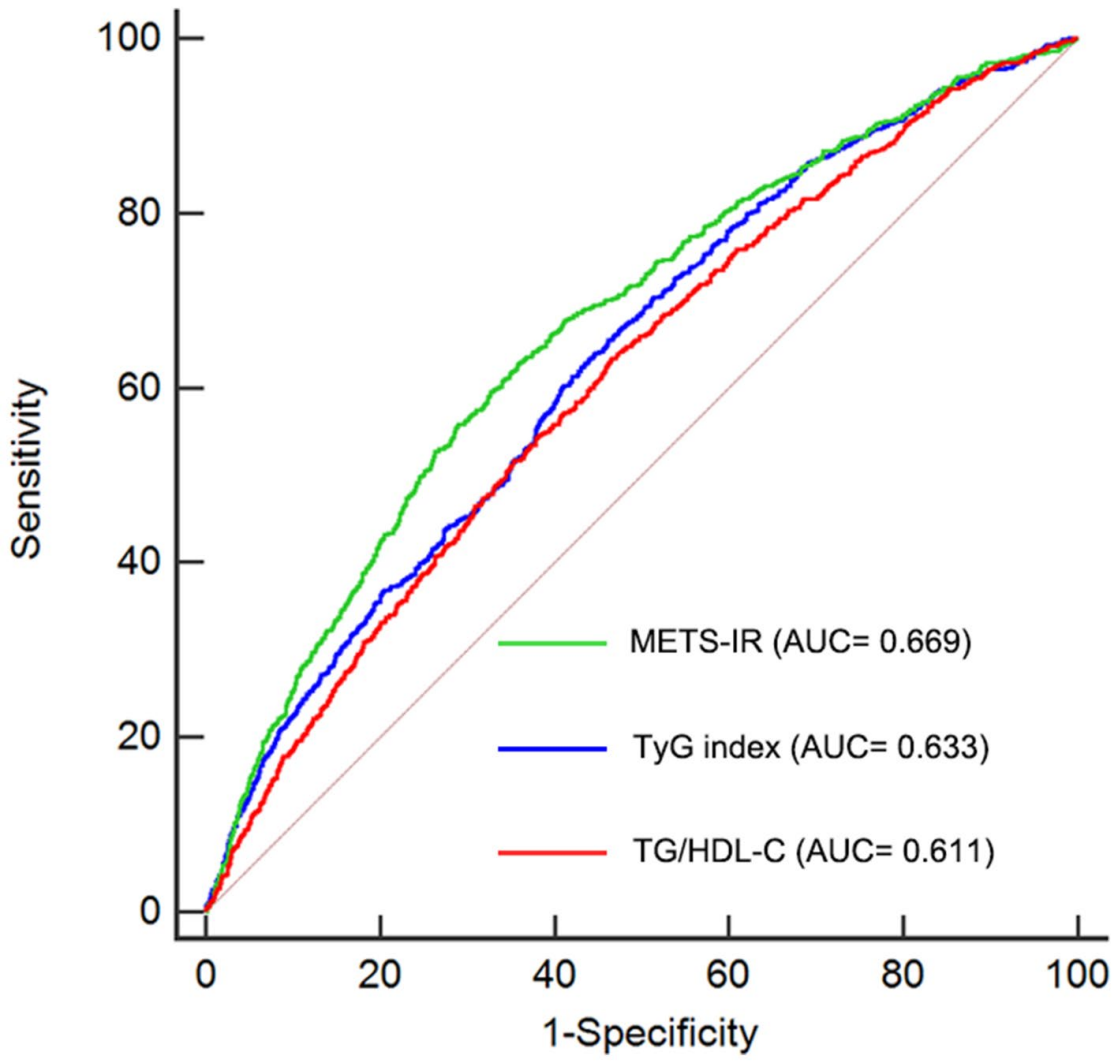
Receiver-operating characteristic curves for prediction of CMM. AUC, area under the curve; CMM, cardiometabolic multimorbidity; TG/HDL-C, triglyceride to high-density lipoprotein cholesterol ratio; TyG index, triglyceride glucose index; METS-IR, Metabolic score for insulin resistance.

Previous studies have identified several other alternative indicators of non-insulin–based IR, including the ratio of TG to HDL-C (TG/HDL-C) and triglyceride glucose index (TyG index) (23, 24). In this study, we conducted ROC analysis to compare the predictive abilities of TG/HDL-C, TyG index, and METS-IR. As shown in Table 4 and Figure 5, the average AUCs for TG/HDL-C, TyG index, and METS-IR were 0.611 (95% CI = 0.600–0.623), 0.633 (95% CI = 0.621–0.644), and 0.669 (95% CI = 0.657–0.680), respectively. The METS-IR demonstrated significantly higher AUCs for predicting the risk of CMM compared to TG/HDL-C (*p* < 0.001) and TyG index (*p* < 0.001).

**Table 4:** Analysis of the ROC curve for predictive power of CMM.

	AUC	SE	95% CI
TG/HDL-C	0.611	0.0107	0.600–0.623
TyG index	0.633	0.0106	0.621–0.644
METS-IR	0.669	0.0105	0.657–0.680

ROC, receiver operating characteristic; TG/HDL-C, triglyceride to high-density lipoprotein cholesterol ratio; TyG index, triglyceride glucose index; METS-IR, Metabolic score for insulin resistance; CMM, cardiometabolic multimorbidity; AUC, area under curve; SE, standard error; CI, Confidence interval.

## Discussion

To the best of our knowledge, this study is the first to explore the association between METS-IR and the risk of developing CMM using a nationally representative prospective cohort. Our study showed a significantly positive and nonlinear association between the METS-IR and CMM regardless of adjustment for other confounding factors. Furthermore, our findings also provided evidence that METS-IR had better discrimination ability in predicting the incidence of CMM than other non-insulin-based IR indexes including TG/HDL-C and TyG index.

IR is defined as the impaired biologic response of target tissues to insulin stimulation (15). Increasing evidence suggests that IR is closely associated with type 2 diabetes and can independently predict CVDs (9, 25–27). Recently, a novel non–insulin-based surrogate indicator of IR, known as METS-IR, has gained increasing attention. METS-IR has been proved to be superior to both the TyG index and TG/HDL-C in diagnosing diabetes (14), which was consistent with our results that METS-IR demonstrated significantly higher AUCs for predicting the risk of CMM compared to TG/HDL-C and TyG index. Additionally, previous studies have demonstrated a close association between METS-IR and various CVDs. In a prospective study involving 2,533 consecutive participants undergoing PCI, METS-IR was found to be associated with the major adverse cardiac and cerebrovascular events (28). A study by Xu et al. revealed that METS-IR was linked to the risk of incident hypertension (29). Qian et al. identified METS-IR as a predictor of heart disease and stroke in the middle-aged and elderly population in China (30). Furthermore, METS-IR was proved to be a promising novel index for predicting the risk of heart failure (16). Additionally, previous studies have demonstrated that METS-IR was linked to the risk of Type 2 Diabetes and could evaluate the cardiometabolic risk (14, 31). Similarly, our study also demonstrated a strong association between elevated METS-IR levels and an increased risk of CMM among middle-aged and elderly populations in China, corroborating the findings of previous research. However, no studies have investigated the predictive value of METS-IR for CMM risk, as previous studies have primarily focused on individuals CMD. In the present study, we demonstrated for the first time that higher METS-IR was significantly associated with increased risk of CMM in a large-scale, national prospective cohort study.

Cardiometabolic multimorbidity (CMM) is defined as the simultaneous presence of at least two cardiometabolic diseases, including diabetes, heart disease and stroke (32). Previous studies have identified numerous risk factors for CMM. For instance, a study by Ye et al. found that the Chinese visceral adiposity index was linked to the risk of CMM (33). However, they did not group the population according to the types of CMD, which would have provided more detailed information. In the present study, we found that individuals in METS-IR quantiles Q4 were significantly correlated with higher risk of CMM in groups without any CMD, with heart disease, and with diabetes at baseline, compared to those in Q1. However, no association between METS-IR and CMM was observed in group with stroke at baseline, probably due to the limited number of participants with stroke. A cohort study including 7,970 participants from CHARLS found that a high TyG index was associated with an increased risk of incident CMM (32). In our study, the HRs of CMM reached 4.14 for participants in the quartiles Q4 compared to those in quartiles Q1 without adjustment, which is higher than that reported in above study. Furthermore, METS-IR was proved to be superior to TyG index in predicting the risk of CMM in our study. Additionally, a study by Behnam et al. identified elevated triglyceride, VLDL-C, total cholesterol/HDL-C, TG/HDL-C, and apoB/apoA1 as the predictors of CMM. In their study, only 1,728 male participants were included. Instead, our study encompassed a larger population that included both males and females, thereby enhancing the reliability of our findings. Overall, our results extend the application of METS-IR and fills the gaps in previous research, suggesting that the METS-IR might be a promising indicator for predicting CMM.

Several potential mechanisms may explain the association between METS-IR and the risk of CMM. First, IR can lead to dyslipidemia, visceral obesity, elevated inflammatory markers and reactive oxide species, each of which is an independent risk factor for CVDs (34, 35). Second, elevated insulin can disrupt fibrinolysis by increasing the circulating concentration of plasminogen activator inhibitor 1. This alteration may result in a pro-thrombotic environment, thereby enhancing platelet aggregation within the cardiovascular system (28). Third, inappropriate activation of the renin-angiotensin-aldosterone system by IR contributes to water and sodium retention and high blood pressure, ultimately increasing the risk of cardiovascular and cerebrovascular disease (36). Finally, the similar dietary behaviors and lifestyle among individuals with IR and CMM may partially account for this association, including excessive high-fat food intake and lack of exercise (35, 37, 38).

The present study is based on data from the China Health and Retirement Longitudinal Study, which is a large-scale and nationally representative prospective cohort. This study effectively adjusted for confounding factors and evaluated the nonlinear relationship between METS-IR and the risk of CMM using RCS analysis, thereby enhancing the reliability of the results. However, there are several limitations that should be acknowledged. First, our study focused mainly on individuals aged ≥45 years in China, and the generalizability to young persons and other races may be limited. Second, our focus was solely on baseline METS-IR level, disregarding its dynamic change that could have provided valuable insight into the underlying mechanism. Third, CMM was determined according to a self-reported physician diagnosis, which might have led to information bias. In the future, large-scale, randomized controlled trials are needed to confirm our findings. Forth, excluding participants with missing METS-IR values as well as those without complete CMM data and lost to follow up could introduce the selection bias. But ultimately, our study included a large enough population to mitigate the effects of selection bias to some extent. Finally, some confounding factors, including the use of lipid-lowering medications, were not accounted for due to data limitations. Future study is needed to pay more attention to the collection and analysis of relevant data.

## Conclusion

In the present study, we found that a higher METS-IR was significantly associated with an increased risk of CMM. Our findings underscore the importance of maintaining low level of METS-IR to reduce the risk of CMM. This has substantial implications for clinical practice and epidemiological research.

## Data Availability

The original contributions presented in the study are included in the article/Supplementary material, further inquiries can be directed to the corresponding author.

## Footnotes

^1^ https://medsta.cn/software
