# Supplementary material for: Relationship of METS-IR with cardiometabolic multimorbidity in China: a nationwide longitudinal cohort study
Source: Front Nutr. 2025 Feb 12;12:1518840. doi: 10.3389/fnut.2025.1518840 (PMC11860098; doi:10.3389/fnut.2025.1518840)
Supplement: Supplementary file 3 [file Data_Sheet_1.ZIP › Code-R.docx]

Dear editor, the followings are the R language code for logistic regression analysis and cox proportional hazards regression:

**Code for logistic regression:**

> library(readxl)

> library(dplyr)

> library(broom)

> library(ggplot2)

> Workbook <-"D:/KM.xlsx"

> file_path <- "D:/KM.xlsx"

> data <- read_excel(file_path)

> data$metsIR四分组 <- as.factor(data$metsIR四分组)

> logistic_model <- glm(CMM是否 ~ metsIR四分组, data = data, family = binomial)

> summary(logistic_model)

> exp(cbind(OR = coef(logistic_model), confint(logistic_model)))

**Code for cox proportional hazards regression:**

library(survival)

library(survminer)

> Workbook <-"D:/KM.xlsx"

> file_path <- "D:/KM.xlsx"

> data <- read_excel(file_path)

> data$metsIR四分组 <- as.factor(data$metsIR四分组)

> surv_object <- Surv(time = data$TIME, event = data$events)

> cox_model <- coxph(surv_object ~ metsIR四分组, data = data)

> summary(cox_model)

> hr <- exp(coef(cox_model))

> ci <- exp(confint(cox_model))

> results <- data.frame(Hazard_Ratio = hr, Lower_CI = ci[,1], Upper_CI = ci[,2])

> print(results)

For restricted cubic splines analysis, we used an online website called I STATISTICS to draw RCS graphs and analyze them (https://medsta.cn/software). This platform is an online statistical analysis platform launched by Professor Zheng Weijun's team from the Teaching and Research Department of Medical Statistics of Zhejiang Chinese Medicine University based on R language. Kaplan-Meier survival curve was conducted using SPSS 26.
